# Chromosome-level genome assembly of the hamour (orange-spotted grouper), *Epinephelus coioides*

**DOI:** 10.12688/f1000research.153918.1

**Published:** 2025-10-29

**Authors:** Razan Khalifa, Tim Bean, Dana Albatesh, Ronny van Aerle, Zenaba Kahtir, Zainab Hizam, Marta Gut, Francisco Câmara, Fernando Cruz, Jèssica Gómez-Garrido, Tyler Alioto, Eduarda Santos, Alexandra Leitão, Diana Minardi

**Affiliations:** 1 Environmental Science Center, Qatar University, Doha, Qatar; 2 University of Exeter College of Life and Environmental Sciences, Exeter, England, UK; 3 The University of Edinburgh The Roslin Institute, Roslin, Scotland, UK; 4 Centre for Environment Fisheries and Aquaculture Science, Weymouth, England, UK; 5 Centro Nacional de Análisis Genómico, Barcelona, Spain; 6 Universitat de Barcelona, Barcelona, Spain

**Keywords:** Aquaculture, Nanopore, Illumina, Arabian Gulf, teleost, Serranidae, Qatar, whole genome sequencing

## Abstract

We present a chromosome-level genome assembly and annotation of the hamour, or orange-spotted grouper ( *Epinephelus coioides*), a high-value and significant teleost fish species across West Indo-Pacific regions of the Middle East, South Africa, and Australia. This species is a popular target for both commercial and recreational fishing and it is widely cultured around the world, particularly in the Asia-Pacific region. The hamour genome was sequenced from one individual male originating from a wild population in the Arabian Gulf and assembled into a 1.07 Gb assembly, the largest 24 superscaffolds making up 99.9% of the assembly. Annotation of the genome identified 28,384 protein-coding genes, with 98.9% single-copy BUSCO gene completeness (Actinopterygii database). These data will support further studies on functional ecological and evolutionary genomics of this species, enhancing the understanding of its biology and its responses to stressors including pathogens.

## Introduction

*Epinephelus coioides* ( Hamilton, 1822) *,* commonly known as the orange-spotted grouper or hamour in the Arabian Gulf region, is a fish species that belongs to the family Serranidae ( Rimmer and Glamuzina, 2019). It is widely distributed across the Indo-Pacific region, including the coastal waters of the Arabian Gulf, comprising Qatar’s coastal regions. This species is highly valued for its meat quality, is a popular target for both commercial and recreational fishing, and is widely cultured around the world particularly in the Asia-Pacific region ( Ranjan et al., 2017). While hatchery production has been increasing to mitigate the pressures on wild populations ( Antoro et al., 2006), juveniles are still being captured from the wild for mariculture operations ( Tupper and Sheriff, 2008), with potential impacts for wild population sustainability.

The hamour presents a distinctive appearance with an elongated body and a large mouth. Its coloration can vary, but it typically features a brownish or reddish background adorned with prominent orange or reddish spots, giving it its common name. It has a robust build and can reach large sizes, with adult specimens sometimes exceeding one meter in length ( Chen et al., 2005). This species constitutes a highly valued commodity, reaching substantial prices in the international market, for example ranging from 11.70 to 40.30 USD per kilogram with an average of 21.45 USD per kilogram in Hong Kong and south east Asia ( Ranjan et al., 2017). In the Arabian Gulf, it is the most important reef-associated commercial species, collected via demersal traps, longlines, and trawls ( Grandcourt et al., 2009). In northern Oman and Iran, it is targeted using wire basket traps and is also commonly taken as bycatch in trawl fisheries ( McIlwain et al., 2016).

Genome sequencing plays a vital role in understanding the genetic makeup of a species, including both genes and regulatory elements, and facilitates understanding of its evolutionary history. By analysing its genome, insights into multiple aspects of a species’ biology, such as disease resistance, growth patterns, and reproductive characteristics can be obtained. To date, several genomics studies have been performed in this species, for example, genome-wide association studies were used to explore ammonia tolerance ( Xu et al., 2019), a highly flexible and repeatable single nucleotide polymorphism genotyping method was employed to study its growth and ammonia tolerance ( Shan et al., 2023), and whole genome sequencing and analysis revealed key regulatory pathways influencing sex differentiation ( Li et al., 2023).

Here, we present a highly continuous chromosome-level genome assembly of this species, obtained using long and short read sequencing technologies and Omni-C scaffolding.

## Methods

### Sampling and nucleic acids extractions

One individual male hamour (43 cm) was caught from the wild (East Qatar, Arabian Gulf, September 2022), and sampled directly. For genomic DNA extraction, 50 mg of liver tissue were excised, submerged in isopentane bath (prepared over dry ice) until frozen (30 s), stored in a pre-chilled cryotube and preserved at -80 °C until further analyses. In parallel, 50 mg of tissue from liver, kidney, heart, spleen, gonad, muscle, skin, gill, and tail were collected from the same specimen and placed in cryotubes containing RNAlater ^®^ (Sigma-Aldrich), stored at +4 °C for 24 hrs to ensure appropriate tissue preservation, and then moved to -80 °C until RNA extraction.

High molecular weight (HMW) DNA was extracted from the liver sample using QIAGEN ^®^ Genomic tip Blood & Cell Culture DNA Midi Kit (QIAGEN) following the manufacturer’s protocol. Purified DNA was quantified using an Invitrogen™ Qubit™ DNA BR Assay kit (Thermo Fisher Scientific), the purity of the sample was analysed using a NanoDrop™ 2000 spectrophotometer (Thermo Fisher Scientific) UV/Vis, and the integrity of the DNA was assessed with Femto Pulse Genomic DNA 165 kb kit (Agilent). DNA was stored at +4 °C until library preparation.

Total RNA was extracted using a Promega Maxwell ^®^ RSC 48 Instrument and Maxwell RSC simply RNA Tissue kit (Promega), quantified using an Invitrogen Qubit RNA BR Assay kit (Thermo Fisher Scientific), and analysed for purity using an RNA 6000 Nano Bioanalyzer 2100 Assay (Agilent). Purified RNA samples were stored at -80 °C until library preparation.

### Library preparation and sequencing

A long-read library for Oxford Nanopore Technologies™ (ONT) sequencing was prepared from DNA extracted from the liver using the 1D Sequencing kit SQK-LSK110 (ONT). In brief, 3 μg of DNA underwent end-repair and adenylation using the NEBNext ^®^ Ultra™ II End Repair/dA-Tailing Module (New England Biolabs), followed by ligation of sequencing adaptors. The ligation product was purified using Beckman Coulter™ AMPure XP Beads (Beckman Coulter Life Science) and eluted in Elution Buffer (ONT). The library was sequenced on a PromethION™ 24 instrument with a R9.4.1 flow cell, and data collected for 110 hours. The quality parameters of the sequencing run were monitored in real time using the MinKNOW™ platform v22.10.7 (ONT, https://nanoporetech.com/document/experiment-companion-minknow) and basecalling performed using Guppy v6.3.9 (ONT, https://nanoporetech.com/document/Guppy-protocol ).

For the proximity ligation library, the Dovetail ^®^ Omni-C ^®^ Kit (Dovetail Genomics, Cantata Bio) was used on the HMW DNA extracted from the liver, following the manufacturer’s protocol. After reversal crosslinking, the DNA was purified and biotinylated chimeric molecules isolated using streptavidin beads before PCR enrichment with 12 PCR cycles using KAPA HiFi HotStart Ready Mix (Roche). The short-insert paired-end library for whole genome sequencing was prepared using the PCR-free protocol and the KAPA HyperPrep kit (Roche). After end-repair and adenylation, Illumina™ platform-compatible adaptors with unique dual indexes and unique molecular identifiers (Integrated DNA Technologies) were ligated. The sequencing library was quality controlled on a 2100 Bioanalyzer using the DNA 7500 assay (Agilent), quantified with KAPA Library Quantification Kit (Roche), and sequenced on four lanes of a NovaSeq™ 6000 (Illumina) with a read length of 2×151 bp.

Total RNA from individual tissues (tail, gonad, heart, gill, skin, spleen, liver, kidney, and muscle) was used to prepare RNA-Seq tissue-specific libraries with a KAPA Stranded mRNA-Seq kit (Roche) following the manufacturer’s protocol. The transcriptomes were sequenced on 4 lanes of a NovaSeq 6000 (Illumina) with a read length of 2×151 bp.

### Nuclear genome assembly, Omni-C scaffolding, curation, and assembly quality checks

Data generated with the PromethION and NovaSeq 6000 were assembled with the Centro Nacional de Análisis Genómico (CNAG) Snakemake pipeline v2.0 ( https://github.com/cnag-aat/assembly_pipeline) to obtain an optimal base assembly for further Omni-C scaffolding. The list of programs, parameters and versions used to assemble and quality check the genome are presented in Table 1. In brief, Illumina reads were processed with Cutadapt ( Martin, 2011), while ONT reads were filtered with FiltLong ( https://github.com/rrwick/Filtlong). Filtered ONT reads were assembled with both Flye ( Kolmogorov et al., 2019) and NextDenovo ( Hu et al., 2024). GenomeScope2 ( Ranallo-Benavidez et al., 2020; Vurture et al., 2017) was used to estimate genome size with the 20-mers present in the pre-processed Illumina reads. The NextDenovo ( Hu et al., 2024) assembly was polished with both ONT and Illumina paired-end reads using Hypo ( Kundu et al., 2019) and then the polished assembly was collapsed with purge_dups ( Guan et al., 2020) to remove haplotypic duplications.

**Table 1.** Programs with citations, versions and parameters used in the present study. Dark grey: genome assembly. Light grey: genome annotation. Dark blue: assembly checks. Light blue: genome curation.

Program	Version	Parameters ^ and notes
Augustus ( Stanke et al., 2006)	3.5.0	
BEDtools ( Quinlan and Hall, 2010)	2.29.0	
BLAST ( Altschul et al., 1990)	2.12.0	Against UniProt (May 2023)
BlobToolKit ( Challis et al., 2020)	4.1.5	
BUSCO ( Manni et al., 2021)	5.7.1	-m genome (odb_10 Actinopterygii, Fungi, and Bacteria)
Cutadapt * ( Martin, 2011)	4.1	-q 20 --paired --retain_unpaired
Dovetail Genomics https://omni-c.readthedocs.io/en/latest/fastq_to_bam.html		-mq 40
ESPRESSO ( Gao et al., 2024)	1.3.0	
EVidenceModeler ( Haas et al., 2008)	1.1.1	
fasta-stats.py https://github.com/cnag-aat/scripts/blob/main/fasta-stats.py		
FiltLong * https://github.com/rrwick/Filtlong	0.2.1	--min_length 1000 --min_mean_q 80
Flye * ( Kolmogorov et al., 2019)	2.9.1-b1780	--nano-raw -i 2 --scaffold -g 2g
GeneID ( Alioto et al., 2018)	1.4	
Genemark-ET ( Lomsadze et al., 2014)	4.71	
GenomeScope2 * ( Ranallo-Benavidez et al., 2020; Vurture et al., 2017)	2	
Hypo * ( Kundu et al., 2019)	1.0.3	-c 100.62178934949716 -s 600m
merqury * ( Rhie et al., 2020)	1.3	k=19
minimap2 * ( Li, 2018)	2.24-r1122	-ax map-ont
miniprot ( Li, 2023)	0.6	
NextDenovo * ( Hu et al., 2024)	2.5.0	read_cutoff=1k genome_size=600m seed_depth=45 seed_cutoff=0 blocksize=1g
PANNZER ( Törönen and Holm, 2022) http://ekhidna2.biocenter.helsinki.fi/sanspanz/		
PASA ( Haas et al., 2008)	2.5.2	
PretextGraph https://github.com/sanger-tol/PretextGraph	0.0.6	
PretextView https://github.com/sanger-tol/PretextView	0.2.5	
purge_dups * ( Guan et al., 2020)	1.2.5	cutoffs -l 5 -m 192 -u 576
RepeatMasker ( Smith et al., 2007) http://www.repeatmasker.org	4.1.2	
RepeatModeler https://github.com/Dfam-consortium/RepeatModeler	1.0.11	
SAMtools ( Danecek et al., 2021; Li et al., 2009)	1.9	
STAR ( Dobin et al., 2013)	2.7.2a	
StringTie ( Pertea et al., 2015)	2.2.1	
TACO ( Niknafs et al., 2017)	0.7.3	
telomeric-identifier ( Brown et al., 2023)	0.2.41	
TransDecoder https://github.com/TransDecoder/TransDecoder	5.7.1	
YaHS * ( Zhou et al., 2023)	1.2a.2	

* Program ran within the Centro Nacional de Análisis Genómico (CNAG) snakemake pipeline v2.0 ( https://github.com/cnag-aat/assembly_pipeline).

^ If different from default parameters.

For further proximity ligation-based scaffolding, a total of 206.96 million Omni-C read pairs were mapped to the assembled genome using the Dovetail Genomics recommended protocol ( https://omni-c.readthedocs.io/en/latest/fastq_to_bam.html). After excluding PCR duplicates, 106.91 million valid Omni-C read pairs were used to scaffold the assembly with YaHS ( Zhou et al., 2023) using the default initial contig error correction step.

To guide manual curation of the assembly, the ONT read coverage was computed for all positions in the assembly using minimap2 ( Li, 2018), SAMtools ( Danecek et al., 2021; Li et al., 2009), and BEDtools ( Quinlan and Hall, 2010), as well as the location of gaps with fasta-stats.py ( https://github.com/cnag-aat/scripts/blob/main/fasta-stats.py) and telomeres with telomeric-identifier ( Brown et al., 2023). These extensions were added to the contact map using PretextGraph ( https://github.com/sanger-tol/PretextGraph). Manual curation was performed according to the rapid curation protocol from The Sanger Institute ( https://gitlab.com/wtsi-grit/rapid-curation ) using PretextView ( https://github.com/sanger-tol/PretextView). The genome was assessed for completeness with BUSCO using the odb10 Actinopterygii database ( Manni et al., 2021), with Merqury ( Rhie et al., 2020) for consensus accuracy (QV) and k-mer statistics, for contiguity statistics with fasta-stats.py ( https://github.com/cnag-aat/scripts/blob/main/fasta-stats.py), and for contamination with BlobToolKit (with NCBI nt database, August 2023 update) ( Challis et al., 2020) and BUSCO using the odb10 databases for Fungi and Bacteria ( Manni et al., 2021). For comparison with the genome assembled in this study, the genome previously described by Li and colleagues ( Li et al., 2023) and available in the European Nucleotide Archive (accession ID: PRJEB28248) was also assessed for completeness with BUSCO’s odb10 Actinopterygii database ( Manni et al., 2021).

### Genome annotation

The hamour genome assembly annotation was obtained by combining transcript alignments, protein alignments and *ab initio* gene predictions. The list of programs, parameters, and versions used for genome annotation is provided in Table 1. In brief, repeats present in the genome assembly were annotated with RepeatMasker ( Smith et al., 2007; http://www.repeatmasker.org) using the custom repeat library available for *Danio rerio* and a new repeat library specific for this study made with RepeatModeler ( https://github.com/Dfam-consortium/RepeatModeler). After excluding repeats that were part of repetitive protein families from the resulting library, RepeatMasker ( Smith et al., 2007; http://www.repeatmasker.org) was run again with this new library performing a BLAST ( Altschul et al., 1990) search against UniProt (May 2023, https://www.uniprot.org/) to annotate the specific repeats. RNA-seq reads were aligned to the previously assembled genome using STAR ( Dobin et al., 2013). Transcript models were subsequently generated using StringTie ( Pertea et al., 2015) and merged using TACO ( Niknafs et al., 2017). High-quality junctions to be used during the annotation process were obtained by running ESPRESSO ( Gao et al., 2024) after mapping with STAR ( Dobin et al., 2013). Finally, assembled spliced alignments were produced with PASA ( Haas et al., 2008). TransDecoder ( https://github.com/TransDecoder/TransDecoder) was run on the spliced alignments in PASA ( Haas et al., 2008) to detect coding regions in the transcripts. The complete proteomes of *Gymnodraco acuticeps*, *Sander lucioperca*, *Cottoperca gobio*, and *Perca fluviatilis* were downloaded from UniProt (May 2023, https://www.uniprot.org/) and aligned to the genome using miniprot ( Li, 2023). *Ab initio* gene predictions were performed on the repeat-masked assembly with GeneID ( Alioto et al., 2018) and Augustus (with human parameters) ( Stanke et al., 2006), and Genemark-ET in self-trained mode ( Lomsadze et al., 2014) with and without incorporating evidence from the RNA-seq data. Finally, all the data were combined into consensus coding sequence models using EVidenceModeler ( Haas et al., 2008). Additionally, untranslated regions (UTRs) and alternative splicing forms were annotated via two rounds of PASA ( Haas et al., 2008) annotation updates. Functional annotation was performed on the annotated proteins with PANNZER’s online server ( Törönen and Holm, 2022; http://ekhidna2.biocenter.helsinki.fi/sanspanz/).

## Results, Discussion, and Conclusions

**Figure 1.**
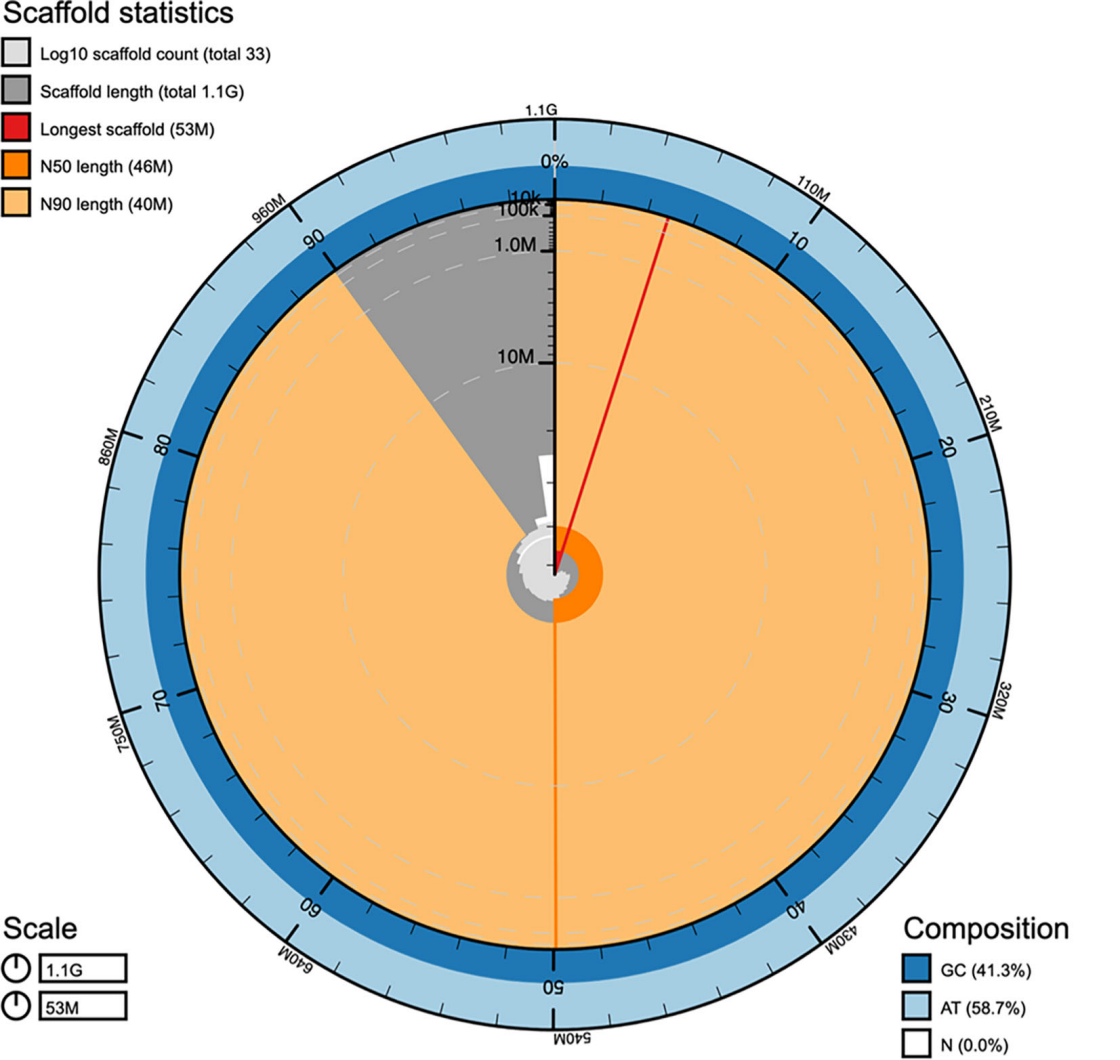
Snail plot summary of assembly statistics for the hamour genome assembly produced in this study. The main plot is divided into 1,000 size-ordered bins around the circumference with each bin representing 0.1% of the 1,071,864,792 bp assembly. The distribution of scaffold lengths is shown in dark grey with the plot radius scaled to the longest scaffold present in the assembly (52,562,209 bp, in red). Orange and pale-orange arcs show the N50 and N90 scaffold lengths (45,643,039 and 39,861,643 bp respectively). The light grey spiral shows the cumulative scaffold count on a log scale with white scale lines showing successive orders of magnitude. The blue and light-blue area around the outside of the plot shows the distribution of bases GC, AT, and N % in the same bins as the inner plot. bp: base pair; GC: guanine-cytosine; AT: adenine-thymine; N: nucleobase.

ONT whole genome sequencing produced 137.75 Gb of data (coverage=128.62x) and Illumina produced 67.73 Gb of 2x151 bp pair-end reads (coverage=63.24x). Genome size (genome haploid length) estimated with GenomeScope2 ranged from 1,088,845,762 to 1,089,817,901 bp ( Table 2). For proximity ligation-based scaffolding, a total of 206.96 million Omni-C read pairs were mapped to the intermediate assemblies generated with NextDenovo ( Hu et al., 2024), resulting in a final assembly with scaffold N50 of 45.64 Mb, N90 of 39.86 Mb and accounting for 1.07 Gb ( Table 3, Figure 1), consistent with the GenomeScope2 ( Ranallo-Benavidez et al., 2020; Vurture et al., 2017) estimation. The assembled genome consists of 24 superscaffolds (making up 99.9% of the assembly) in accordance with the previously reported diploid karyotype (2n=48) for this species ( Wang et al., 2010). It had a consensus accuracy of QV=47 and single-copy BUSCO gene completeness of 98.9% (BUSCO odb10 Actinopterygii) ( Table 3). No evidence of contamination was detected. Together, these statistics indicate that we have assembled a high quality, chromosome-level genome for the hamour.

**Table 2.** Hamour genome assembly size. Genome size estimated by GenomeScope2 ( Ranallo-Benavidez et al., 2020; Vurture et al., 2017) on the pre-processed Illumina reads. bp: base pairs.

Attribute	Minimum	Maximum
Homozygous	99.64%	99.66%
Heterozygous	0.34%	0.36%
Genome Haploid Length (bp)	1,088,845,762	1,089,817,901
Genome Repeat Length (bp)	320,257,215	320,543,145
Genome Unique Length (bp)	768,588,547	769,274,755
Model Fit	74.61%	98.69%
Read Error Rate	0.14%	0.14%

**Table 3.** Hamour genome assembly results and comparison with current publicly available genome ( Li et al., 2023). Genome completeness was assessed with BUSCO ( Manni et al., 2021) using the Actinopterygii odb10 database updated on the 08/01/2024. Number of BUSCO groups searched for in the Actinopterygii database was 3,640. bp: base pairs; Mb: megabases.

Attribute	This study	Li et al., 2023
Genome assembly total length (bp)	1,071,864,792	1,023,559,032
Scaffolds number	33	1450
Scaffold N50 (Mb)	45	2
Contigs number	140	159
Contig N50 (Mb)	18	2
Completeness	99.3%	99.0%
Single-copy	98.9%	98.5%
Duplicated	0.4%	0.5%
Fragmented	0.6%	0.7%
Missing	0.1%	0.3%

A comparison of the chromosome-level genome assembly produced in this study and a previously published genome ( Li et al., 2023) is presented in Table 3. Genome size was consistent in both studies (1.07 and 1.02 GB in the present and previous study, respectively). Our assembly had a lower number of scaffolds with a greater scaffold length, providing an improvement on the previously published genome and contributing to the advancement of research for this species.

The genome annotation identified 28,384 protein-coding genes, producing 39,296 transcripts (1.38 transcripts per gene), which improved on the number of annotated protein coding genes reported in Li et al. (2023) (26,931). The annotated transcripts contained 11.05 exons on average, with 91% of them being multi-exonic ( Table 4).

**Table 4.** Genome annotation results. The hamour genome assembly annotation was achieved by combining transcript alignments, protein alignments and *ab initio* gene predictions. The genome was annotated with 28,384 protein-coding genes, producing 39,296 transcripts (1.38 transcripts per gene). bp: base pairs; Mb: megabases.

Attribute	Result
Number of protein-coding genes	28,384
Median gene length (bp)	8,711
Number of transcripts	39,296
Number of exons	284,015
Number of coding exons	269,906
Median UTR length (bp)	752
Median intron length (bp)	500
Exons/transcript	11.052
Transcripts/gene	1.38
Multi-exonic transcripts	91%
Gene density (gene/Mb)	26.48

Here we report on the sequencing and assembly of a hamour individual from the Arabian Gulf using a combination of Nanopore and Illumina sequencing technologies. We produced a chromosome-level assembly for this species and have improved on its annotation compared to a previously released genome. The genome sequence, raw data, and annotation are released openly for reuse. All raw sequence data, the assembly, and annotations have been deposited in INSDC databases, with accession identifiers reported in Table 5. These data will facilitate further studies on the biology of this species and on its management in the wild and aquaculture settings.

**Table 5.** Genome data for *Epinephelus coioides* (orange-spotted grouper, hamour).

**Project accession data**	
Assembly identifier	QU_Ecoi
Species	*Epinephelus coioides*
Specimen	QU-Ecoi-1
NCBI Taxonomy ID	94232
BioProject	PRJNA1128520
BioSample ID	SAMN42050860, SAMN43492902-SAMN43492913
Isolate information	QU-Ecoi-1
**Raw data accessions**	
Oxford Nanopore PromethION	SRR30574011
Omni-C Illumina	SRR30574012
Illumina short-read	SRR30574003
Illumina RNASeq	SRR30574004-SRR30574010; SRR30574013-SRR30574014
**Genome assembly**	
Assembly accession	GCA_051314025.1

## Ethical considerations

Due the nature of the research project, with no experimental work on live animals (working only with tissues collected from dead animals), an exemption certificate from our institutional animal care and use committee (IACUC) was obtained for the use of wild fish caught by independent fishermen and bought by the author immediately after capture. The hamour used in this study was euthanised via immersion in MS-222 and destruction of the brain immediately after being caught. This study followed all relevant ethical guidelines and protocols approved by the institutional animal care and use committee (IACUC).

## Data Availability

The assembled genome and raw data are publicly available through the National Centre for Biotechnology Information (NCBI) and Short Read Archive (SRA) under the following identifiers: Organism: *Epinephelus coioides* QU-Ecoi-1, Assembly accession: GCA_051314025.1, BioProject: PRJNA1128520, and BioSample: SAMN42050860 ( Table 5).
