# Supplementary material for: Chromosome-level genome assembly of the hamour (orange-spotted grouper), Epinephelus coioides
Source: F1000Res. 2025 Oct 29;14:1180. [Version 1] doi: 10.12688/f1000research.153918.1 (PMC12648030; doi:10.12688/f1000research.153918.1)
Supplement: Peer review history

# Reviewer report: Reviewer response for version 1

**DOI:** 10.5256/f1000research.168876.r428838

**Published:** 2025-12-04

**Authors:** Karim Gharbi

**Affiliations:** 1 The Earlham Institute, Norwich, UK

This genome note reports on the assembly and annotation a chromosome-level reference assembly for the orange-spotted grouper, a popular serranid fish distributed across the Indo-Pacific, and locally known as hamour in the Arabian Peninsula and Persian Gulf region. The note includes a high-level introduction of the species followed by assembly/annotation methods and statistics, with comparison to a previously published assembly from the same species.

 The introduction highlights the importance of the species for both commercial and recreational fishers, with a growing aquaculture sector. The sequencing, assembly, and annotation methods are comprehensive and generally follow established practices.The assembly report shows that the authors have achieved chromosome-level scaffolds and significantly improved on the quality of the previous assembly. They have also improved on the number of identified protein-coding genes relative to the previous annotation. 

 This study represents a valuable contribution to the field of fish genomics, and more specifiocally species targeted by aquaculture. The availability of a chromosome-level assembly for the hammer will likely have a significant impact on our understanding of the species biology, including conversation and sustainable exploitation. 

 Below are specific comments, which the authors may want to consider to clarify and improve the manuscript. 

 Main comments

 1. Please comment on the recovery and assembly of the mitrochondrial genome.

 2. Please clarify that the starting material for Dovetail Omni-C library preparation (currently listed as HMW DNA). 

 3. Please specify which base-calling model was used in Guppy v6.3.9.

 4. Please indicate the type and number of changes introduced during manual curation.

 Minor commetns

 1. Please indicate whether the specimen has been vouchered and include a digital image. 

 2. Please clarify that the Bioanalyzer assay was used to check the intergrity of the RNA extraction.

Are the datasets clearly presented in a usable and accessible format, and the assembly and annotation available in an appropriate subject-specific repository?

Yes

Are sufficient details of the sequencing and extraction, software used, and materials provided to allow replication by others?

Partly

Are the rationale for sequencing the genome and the species significance clearly described?

Yes

Are the protocols appropriate and is the work technically sound?

Yes

Reviewer Expertise:

Genomics, bioinformatics

I confirm that I have read this submission and believe that I have an appropriate level of expertise to confirm that it is of an acceptable scientific standard, however I have significant reservations, as outlined above.

# Reviewer report: Reviewer response for version 1

**DOI:** 10.5256/f1000research.168876.r430760

**Published:** 2025-11-25

**Authors:** Merly Escalona

**Affiliations:** 1 University of Connecticut Department of Molecular and Cell Biology, Storrs, Connecticut, USA

The authors present a set of genomic resources for  the orange-spotted grouper. These resources include a chromosome-level genome assembly which metrics meet the benchmark of a high-quality genome, and its corresponding gene annotation.  

 The genome assembly generated is an improvement on existing genomic resources for the grouper, and extremely welcomed given the evident application of the resource.

 The manuscript is overall it is well written and organized as a the type of manuscript submitted, methods are well described and on par with the current pipelines of genome assemblies for the sequencing technologies used in the study. 

 Having said that, there are a few points in which this manuscript could be improved. 

 In the introduction, the last two paragraphs are a somewhat disconnected. These should be re-organized and combined, or expanded. 

 Given the sequencing data generated, it was expected that the nuclear genome assembly was accompanied by a mitochondrial genome as well. Please think about adding this too to the genomic resources.

 Information about sequencing output, in particular for long-read data, should be presented with a read distribution and a read length vs. quality plot. These plots are more informative than the single amount of output.

 There's no explanation on how k-mer counts were generated for GenomeScope2 and QV metrics.

 The assembled genome consists of many scaffolds, but the largest 24 (considered superscaffolds) make up 99.9% of the assembly.  The phrase as it is written in the manuscript can be misleading.

 You are considering this a chromosome-level assembly, and it has been made available on the INSDC Databases as such. The chromosome naming is not based on ordered sequence length, what's the convention used for the chromosome naming/assignment?  was there a specific genome used as reference for the chromosome naming? if so, which one? Please be explicit.

Are the datasets clearly presented in a usable and accessible format, and the assembly and annotation available in an appropriate subject-specific repository?

Yes

Are sufficient details of the sequencing and extraction, software used, and materials provided to allow replication by others?

Partly

Are the rationale for sequencing the genome and the species significance clearly described?

Yes

Are the protocols appropriate and is the work technically sound?

Yes

Reviewer Expertise:

Genome assembly, comparative genomics, bioinformatics

I confirm that I have read this submission and believe that I have an appropriate level of expertise to confirm that it is of an acceptable scientific standard.

# Reviewer report: Reviewer response for version 1

**DOI:** 10.5256/f1000research.168876.r430757

**Published:** 2025-11-19

**Authors:** Jayan Duminda M Senevirathna

**Affiliations:** 1 Uva Wellassa University, Badulla, Uva Province, Sri Lanka

The article is well written, and the content is clearly explained. The availability of raw data adds to the transparency of the study, and the originality of the work is commendable. However, the following comments are for further improvement.

 Typically, six or fewer keywords are recommended. Please verify and adjust the number of keywords according to the journal’s guidelines.

 The Introduction could be strengthened by including additional background information about the fish species, such as sexual dimorphism, ecological significance, and other relevant biological characteristics.

 In the Methodology section, please specify that the wild fish samples were obtained from a fisherman. This detail will improve the clarity and reproducibility of your sampling procedure. If possible, include the Mausam voucher number. 

 If this study presents a chromosome-level assembly, please include a table summarizing the identified chromosomes, along with relevant details such as chromosome length, number of scaffolds anchored, N50 values, and any other assembly statistics that support the quality of the chromosomal organization. Otherwise, change the topic.

Are the datasets clearly presented in a usable and accessible format, and the assembly and annotation available in an appropriate subject-specific repository?

Yes

Are sufficient details of the sequencing and extraction, software used, and materials provided to allow replication by others?

Yes

Are the rationale for sequencing the genome and the species significance clearly described?

Yes

Are the protocols appropriate and is the work technically sound?

Yes

Reviewer Expertise:

Aquatic Biotechnology and Genetics

I confirm that I have read this submission and believe that I have an appropriate level of expertise to confirm that it is of an acceptable scientific standard.
